# Generation of a new transgenic mouse model for assessment of tau gene silencing therapies

**DOI:** 10.1186/s13195-016-0202-1

**Published:** 2016-09-05

**Authors:** Susan Fromholt, Christian Reitano, Hilda Brown, Jada Lewis, David R. Borchelt

**Affiliations:** 1 Department of Neuroscience, Center for Translational Research in Neurodegenerative Disease, University of Florida, Gainesville, FL USA; 2 McKnight Brain Institute, University of Florida, Gainesville, FL 32610 USA

**Keywords:** Alzheimer’s disease, Tau, Gene-silencing, Transgenic mouse model

## Abstract

**Background:**

Targeting the expression of genes has emerged as a potentially viable therapeutic approach to human disease. In Alzheimer’s disease, therapies that silence the expression of tau could be a viable strategy to slow disease progression.

**Methods:**

We produced a novel strain of transgenic mice that could be used to assess the efficacy of gene knockdown therapies for human tau, in live mice. We designed a tetracycline-regulated transgene construct in which the cDNA for human tau was fused to ubiquitin and to luciferase to create a single fusion polyprotein, termed TUL.

**Results:**

When expressed in brain, the TUL polyprotein was cleaved by ubiquitin-processing enzymes to release the luciferase as an independent protein, separating the half-life of luciferase from the long-lived tau protein. Treatment of bigenic tTA/TUL mice with doxycycline produced rapid declines in luciferase levels visualized by in vivo imaging and ex vivo enzyme measurement.

**Conclusions:**

This new mouse model can be used as a discovery tool in optimizing gene targeting therapeutics directed to reduce human tau mRNA levels.

## Background

Mutations in tau cause familial frontotemporal dementia (FTD-tau) and aggregates of wild-type tau are a prevalent pathology of Alzheimer’s disease (AD) and other sporadic tauopathies [1]. Although tau has clearly been a target of interest in these diseases, there have been relatively few good leads for small molecule therapeutics to reduce tau pathology and slow disease progression. Dominantly inherited familial neurodegenerative diseases, such as FTD, are prime candidates for biologic therapies (biotherapies) in which expression of the mutant gene is directly targeted by gene silencing approaches. Multiple approaches to knocking down mutant gene expression have been described in the literature including viral vector delivery of shRNAi or microRNA mimics, delivery of naked RNAi and RNAi complexed with various reagents to facilitate uptake, and delivery of modified antisense DNA oligonucleotides (ASOs) [2–5]. Preclinical testing of such therapies has met with encouraging outcomes, with examples of good to moderate efficacy [6–9].

The accumulation of misfolded tau appears to correlate well with disease progression in human AD [10] and there are ample data to suggest that patients with high amyloid burden and mild memory impairment would be high risk for subsequent development of tauopathy and further degeneration [11, 12]. Second, the clinical course of AD is relatively slowly progressing, offering a larger window for therapeutic intervention. Third, familial cases of FTD-tau present a highly motivated patient population in which silencing mutant tau expression would appear to be a highly relevant target. Furthermore, FTD represents a particularly attractive target for gene knockdown as it may be possible to direct such strategies against a specific isoform of tau [four repeat (4R)] which is either elevated or preferentially aggregated in many cases of the familial disease [13]. Fourth, although multiple anti-amyloid therapies have reached the human clinic (with limited success so far), the pipeline for modulators of tau biology has been limited [12, 14, 15]. Thus, despite the hurdles created by the need to use invasive procedures to deliver these therapeutics and the difficulties in assessing target engagement in human trials, there is a strong rationale for pursuing gene-silencing therapies for tau in FTD and possibly AD.

In FTD cases caused by mutations in tau, it might be possible to use allele-specific targeting to diminish the levels of mutant tau. Such strategies might be very effective in cases involving mutations that alter tau splicing. In AD cases, however, there are no mutations or obvious splicing alterations and thus a gene silencing approach would target wild-type tau. A recent study to target tau expression in adult mice has demonstrated the potential efficacy of antisense-oligonucleotide gene-silencing approaches and demonstrated that reductions of tau in the adult nervous system may not produce adverse effects [16]. However, it should be noted that humans that are heterozygous for a microdeletion of chromosome 17, which includes the *MAPT* locus and at least two other genes, have multiple developmental defects including mental retardation [17]. Mice lacking *Mapt* have been reported to show defects in neuronal migration [18], and defects in long-term potentiation have been reported in both knockout mice and slice cultures from adult rats treated with tau shRNAi [19]. Although these data suggest a potential for tau-silencing therapies to produce adverse effects in humans, mice with heterozygous or homozygous deletions of tau are cognitively normal, and partial or complete elimination of tau in mice that overproduce human Aβ has been shown to ameliorate Aβ-mediated cognitive deficits [20]. Thus, a gene silencing approach to tau may well be a viable therapeutic strategy for multiple human tauopathies, including AD.

In the present study, we have endeavored to create a new transgenic mouse model expressing the human 4R tau that is designed for the sole purpose of optimizing gene silencing therapies for tau. Optimizing the type of therapeutic, the dose, and the route of delivery for each of these potential therapies in vivo typically involves cross-sectional studies that provide a snapshot of efficacy at whatever times are chosen to sacrifice animals and analyze tissues. In order to more effectively assess the efficacy of these types of therapeutics in vivo, we designed and generated transgenic animals that encode human 4R tau, containing the P301L mutation which is associated with FTD, that is fused in-frame to luciferase for the purpose of generating transgenic mice that could be used to report the efficacy of knockdown therapeutics in a mouse model of tauopathy. By placing the human tau transgene under the control of a doxycycline (Dox) suppressible promoter, we built in an internal positive control into this transgenic model. We demonstrate the utility of this model and the dynamic range of the luciferase reporter as an in vivo indicator of gene expression.

## Methods

### Generation of transgenic mice

**Fig. 1:**
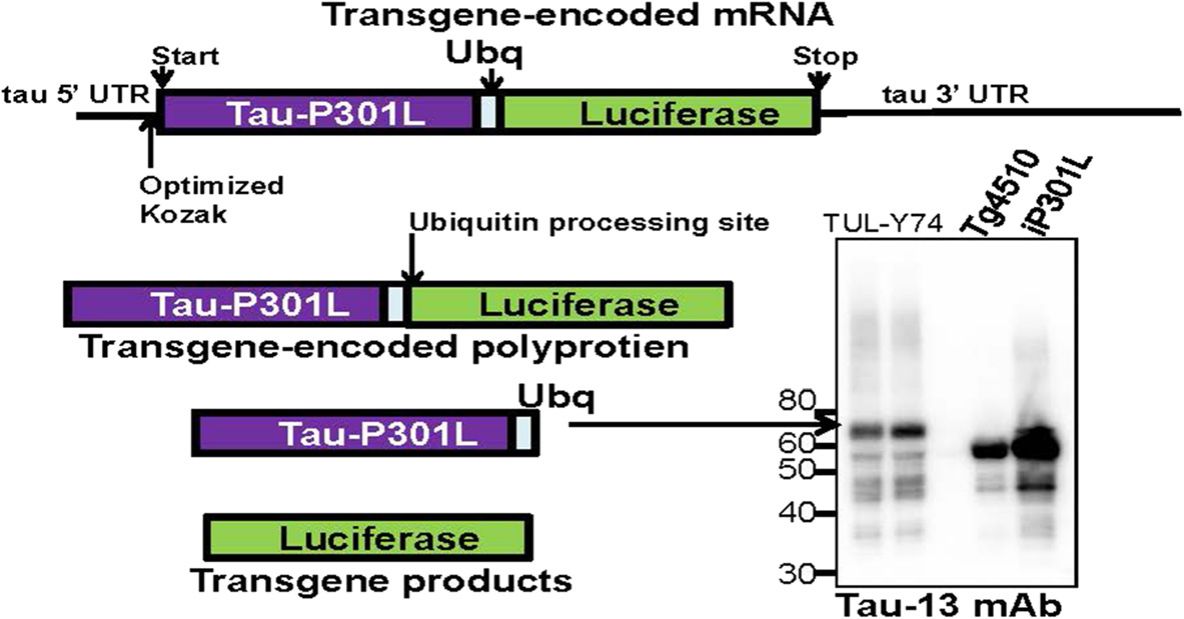
Expression of tau in bigenic tTA/TUL-Y74 mice. The *diagram above the figure* depicts the expected mRNA for the transgene and its products (not precisely to scale). The cDNA for the transgene construct was designed to encode 156 bp of 5′ untranslated sequence and 1227 bp of 3′ untranslated sequence from the human tau gene, providing additional targeting options for knockdown. The levels of tau in the forebrains of two tTA/TUL mice (aged 2 months) are compared to that of Tg4510 P301L tau mice (aged 3 months) and iP301L tau mice (aged 24 months), which express human tau from the same cDNA used in the TUL mice. The blots were probed with a mouse monoclonal antibody that specifically reacts with human tau (clone Tau-13). The tau protein created by processing of the TUL poly-protein would be expected to possess a C-terminal ubiquitin moiety and thus migrate to a slightly higher molecular weight

Transgenic animals were created from transgene vectors based on the tetPrP.*Xho* vector [21, 22]. First, we constructed a cDNA that sequentially encodes tau-ubiquitin-luciferase as a contiguous gene (Fig. 1). The tau gene is a human cDNA for 0N4R tau that encodes the P301L mutation. This cDNA has been expressed in mice previously [23]. The cDNA for ubiquitin is derived from human and the cDNA for luciferase is derived from firefly (*Phontinus pyralis*). The fused cDNA was generated by polymerase chain reaction (PCR)-based methods that amplified each of these genes independently using primers that produced novel restriction endonuclease sites to enable stitching the gene together to create a single open-reading-frame for a poly-protein that we abbreviated as TUL.

After linearizing the vector plasmid with *Not1* enzymes that separate the bacterial plasmid sequences from the promoter element and tetPrP.*Xho* vector sequences, the transgene DNA fragment was purified by agarose gel electrophoresis and injected into fertilized embryos from FVB/NJ mice in the University of Florida Mouse Modeling Core. From these injections, two founders were identified by PCR of the genomic DNA extracted from tail biopsy by standard methods of proteinase K digestion, high salt precipitation, and ethanol precipitation. The PCR protocol used was as follows: ddH_2_O 20.7 μL, 10X PCR buffer (w/MgCl_2_) 2.5 μL, 5 mM dNTPs 0.5 μL, 50 μM PrP-AS (antisense primer -5′-CCA AGC CTA GAC CAC GAG AAT GC-3′), 50 μM TUL-S (sense primer – 5′-CAA ATT GTA ACT CGA TCA GGC CCC TGG GGC GGT-3′), Taq DNA Polymerase (New England Biolabs, Ipswich, MA, USA) 0.1 μL, Tail DNA 1 μL. The PCR program used was as follows: 94 °C for 30 s, 55 °C for 60 s, 72 °C for 3 m, 35 cycles, followed by 72 °C for 10 min then hold at 10 °C.

One of these founders efficiently transmitted the transgene to offspring, producing a line termed Y-74. Mice from this line were crossed to mice that express the tet-Transactivator (tTA) under the CamKIIα promoter [24] [B6.Cg-Tg(Camk2a-tTA)1Mmay/Dbo, Jackson Laboratories, Bar Harbor, ME, USA], which had been backcrossed to FVB/NJ mice twice. Mice bigenic for CamKII-tTA and tet.TUL were expected to express the transgene exclusively in the forebrain.

All procedures involving mice were reviewed and approved by the University of Florida Animal Care and Use Committee. The mice described here are available through the Jackson Laboratories.

### Immunoblotting

To detect transgenic human tau expression in brain, mice were deeply anesthetized and then perfused with ice-cold PBS. The brains were then quickly removed and the forebrains were dissected and flash frozen on dry ice. To homogenize the brain, the tissue was sonicated, using an ultrasonic probe (Misonix microson ultrasonic cell disrupter XL, Farmingdale, NY USA) at an intensity setting of 2. Each brain was sonicated three times for 10 s each in 1× PBS (phosphate-buffered saline) containing 1:100 v/v protease inhibitor cocktail (Sigma, St. Louis, MO, USA). The tissue homogenate was centrifuged at 17,000 × g for 5 min to produce a clarified brain homogenate. The protein concentrations of the resulting homogenates were then determined by bicinchoninic acid assay, as described by the manufacturer (Pierce Biotechnology/Thermo Fisher, Waltham, MA, USA). Fifty micrograms of each brain homogenate were mixed with 2× Laemmli sample buffer and boiled for 5 min. Sample buffers contained β-mercaptoethanol to reduce any disulfide bonds and the samples were electrophoresed in 4–20 % Tris-Glycine sodium dodecyl sulfate-polyacrylamide gels (Invitrogen Life Technologies, Grand Island, NY, USA). Following transfer to nitrocellulose membranes (Amersham/GE Healthcare Life Sciences, Pittsburgh, PA, USA), the membranes were blocked in 5 % milk in PBS-T (1X PBS, 0.1 % Tween-20) for 1 h, then incubated for 2 h at room temperature with the mouse monoclonal antibody Tau 13 (Covance, Princeton, NJ, USA) at 1:4000 in PBS-T and 5 % milk. The membrane was then washed with PBS-T, then incubated for 1 h at room temperature with a goat-mouse secondary antibody (KPL, Gaithersburg, MD, USA) at 1:5000 in PBS-T and 5 % milk before developing with enhanced chemiluminescence reagents (Thermo Scientific Inc., Rockford, IL, USA) and visualizing with a FluorChem E imager (Protein Simple, Santa Clara, CA, USA).

### Imaging

For in vivo imaging of the bigenic tTA/TUL mice, we used the Perkin Elmer IVIS Spectrum In Vivo System (Xenogen Biosciences Perkin Elmer, Waltham, MA, USA). Mice were placed under deep anesthesia and were first imaged prior to injection with luciferin. Mice were then injected with 100 μL of 30 mg/mL XenoLight Rediject D-luciferin Ultra (Xenogen Biosciences Perkin Elmer, Waltham, MA, USA). Images were obtained on the IVIS imager from 6 min after injection (peak signal was previously determined at approximately 12 min after injection of luciferin). During every scan, images were taken at the same exposure setting (determined by the IVIS imager during the first scan) and multiple images were captured to insure that the peak signal had been reached. To quantify the bioluminescence for each animal, we used Living Image Analysis software (Xenogen Biosciences Perkin Elmer, Waltham, MA, USA). With this software, a box was drawn around the area of signal in the region of the head to define a region of interest (ROI). The area of the ROI for each mouse within the same image was equal. The average radiance within the ROI was measured and compared.

### Ex vivo assessment of luciferase activity

Brains were harvested from mice as described above and stored at −80 °C. To prepare lysates, the brains were weighed and then homogenized in PBS (10 volumes relative to mass) as described above to produce a 10 % homogenate. The PBS contained protease inhibitor [1 μL of SIGMA protease inhibitor cocktail (catalog number: P8340-5ML; Sigma-Aldrich, St. Louis, MO, USA) for every 99 μL of PBS 1×]. The mass of the PBS 1× buffer with protease inhibitor was approximated volumetrically by assuming a density of 1 mg/μL at room temperature. To detect luciferase activity, 10 and 20 μL of each sample were dispensed to separate wells in an opaque 96 well plate and 40 and 30 μL of PBS 1× were added to the wells, respectively. Fifty microliters of luciferase substrate solution from the Promega Bright-Glo Luciferase Assay System (catalog number: E2610, Madison, WI, USA) were then added to each well and luminescence values were immediately collected by a plate reader (Biotek Synergy HT Microplate Reader, Winooski, VT, USA).

## Results

Mice from the Y-74 line of TUL mice were crossed to mice that express the tet-Transactivator (tTA) under the CamKIIα promoter to produce mice that exclusively express the TUL construct in forebrain. From the design of the construct, the expectation is that human tau proteins present in these mice would have C-terminal fusion of ubiquitin. Immunoblots of brain homogenates from these mice confirmed the expression of tau with the tau protein migrating at a slightly larger size relative to mice express human tau alone (Fig. 1). The molecular size of firefly luciferase is 550 amino acids – predicted relative molecular weight of 55 kDa. Uncleaved TUL protein would be predicted to migrate at more than 130 kDa. Thus, the tau protein in the TUL mice appears to migrate to a size that would be expected if the luciferase had been cleaved by ubiquitin processing enzymes as envisioned in the design of the construct. Notably, the levels of Tau-Ubq in these mice were lower than the levels detected in two other lines of mice that express human Tau (Tg4510 and iP301L [23]) used here is as positive controls. The responder line of Tg4510 mice is one of the two transgenes that is expressed in the previously described rTg4510 model [23]. In the absence of the tTA transgene, Tg4510 mice express low levels (approximately twofold) of mutant human tau and do not develop tau pathology [23]. The iP301L model is a bigenic model similar to the rTg4510 model that is bigenic for a mutant tau responder transgene and CamKIIα-tTA, resulting in levels of human tau expression that are approximately seven-fold higher than endogenous mouse tau. The levels of mutant human tau in the bigenic tTA/TUL-Y74 mice are lower than that of the Tg4510 responder line and thus would appear to be below the threshold to produce tau pathology.

**Fig. 2:**
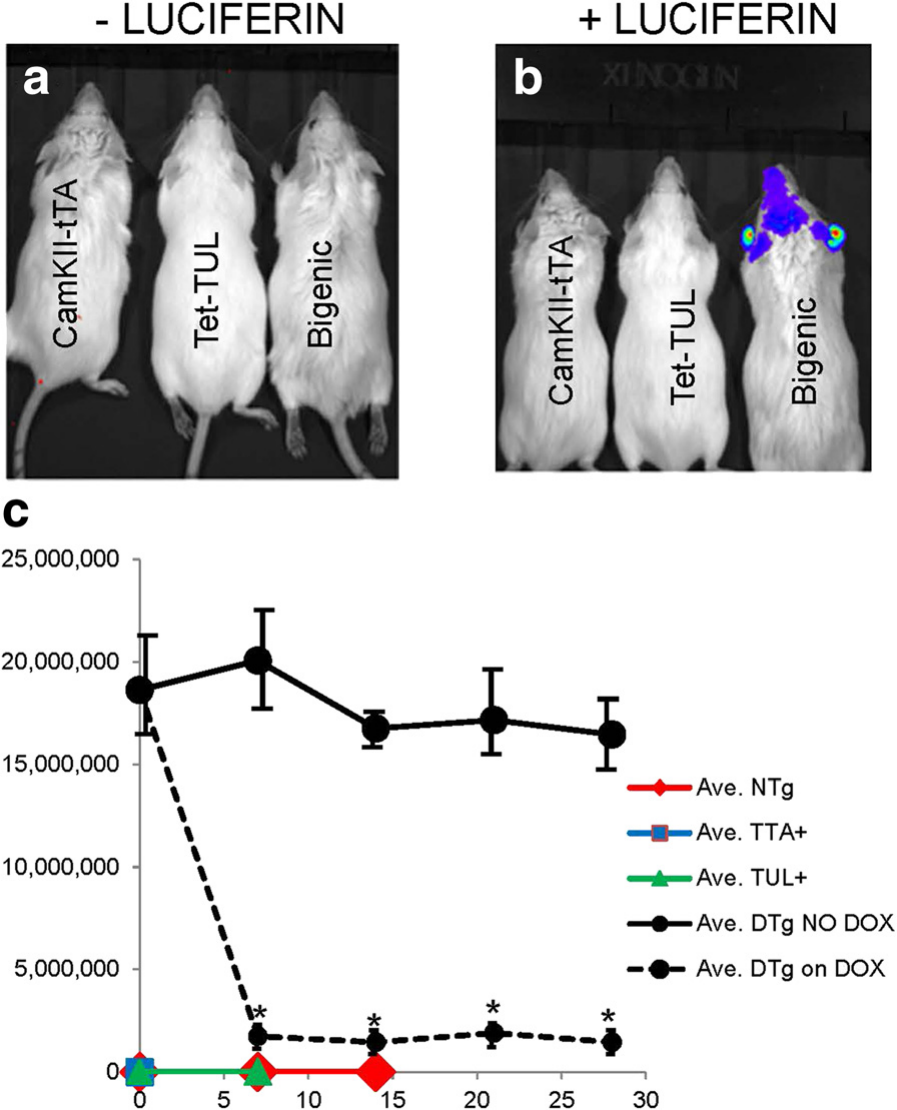
Bioluminescence in bigenic tTA/TUL-Y74 mice. **a** *Baseline images* of three mice of three different genotypes prior to luciferin injection. **b** *Images* of three mice of three different genotypes 10–12 min after luciferin injection. **c** *Graphs* of bioluminescence units in bigenic tTA/TUL-Y74 mice that were either given food containing Dox at day 0 or left untreated. The mice were re-injected and re-imaged at 7, 14, 21, and 28 days. Dox treatment produced sustained suppression of bioluminescence

To determine the utility of these new mice for in vivo imaging, we injected bigenic tTA/TUL mice that were aged 6–7.5 months with the luciferase substrate luciferin. When imaged in an IVIS imaging device, we easily detected bioluminescence in brain (Fig. 2a and b). The levels of luciferase were relatively steady in repeated measures of bioluminescence (Fig. 2c – solid black line) and much higher than the levels transgenic only for the TUL gene (Fig. 2c).

In using the tet-PrP.*Xho *vector to express the TUL construct, we gained two major advantages that make the technology more useable. First, by using CamKII-tTA to drive expression of TUL, we express the protein exclusively in the forebrain and eliminate a potential issue with bioluminescence signal that could come from other sources if the gene were more broadly expressed. Second, we created the possibility for very nice positive controls because the expression of the TUL construct should be suppressed by feeding the mice Dox [21, 23, 24]. To confirm the utility of the model, we conducted a study in which bigenic tTA/TUL mice were followed longitudinally (Fig. 2c, dashed black line). The mice were injected with luciferin, imaged, and put on feed that contains Dox. After 7 days, the mice were imaged again, with repeated imaging at regular intervals. Within the first 7 days, the level of bioluminescence fell by 90 % and remained low as long as the mice were on Dox (re-imaged at 7, 21, and 28 days). When the same mice were put back on regular feed, the levels of brain bioluminescence after injection with luciferin quickly increased (not shown). Together, these data demonstrate that levels of luciferase activity, as measured by in vivo bioluminescence, fall as one would predict after transgene expression is suppressed.

**Fig. 3:**
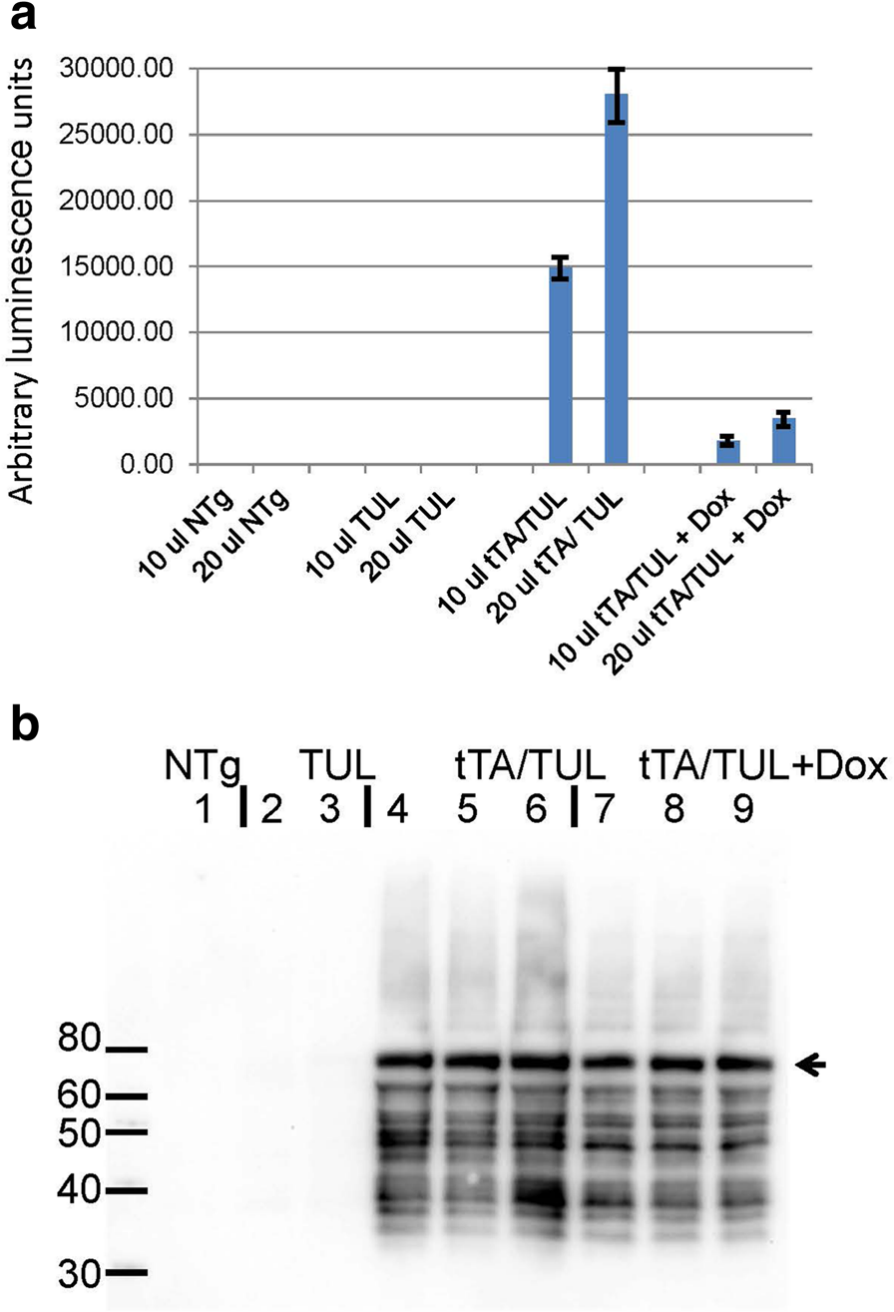
Ex vivo analysis of Luciferase and tau levels. The forebrains of bigenic and single transgenic mice (n = 3 each genotype) were homogenized in PBS (10 % weight/volume). **a** Two different amounts of each homogenate were assayed for luciferase activity as described in “Methods.” Only the bigenic tTA/TUL mice produced high levels of luciferase activity. Bigenic tTA/TUL mice fed chow containing Dox produced 90 % less luciferase activity. The error bar estimates standard deviation. **b** *Immunoblot* of brain homogenates for tau with a monoclonal antibody that specifically reacts with human tau (clone Tau-13). The brain homogenates of three different tTA/TUL mice (no Dox) and three different tTA/TUL mice fed Dox chow for 7 days, along with non-transgenic brain homogenates and mice transgenic for only the TUL responder transgene were analyzed. Each lane contains 50 μg of total protein

To confirm the bioluminescence data, we performed ex vivo analyses of luciferase levels in tissue extracts from these mice (Fig. 3). Brains harvested from mice were homogenized in PBS (10 % weight/volume) and small portions of this homogenate (10 and 20 μL) were assayed for luciferase activity. As expected, the levels of bioluminescence generated by homogenates of NTg and TUL mice lacking the tTA gene were very low (in this assay both were below the level of detection) (Fig. 3a). By contrast, bioluminescence was easily detected in homogenates from tTA/TUL mice with the levels dropping by 90 % in homogenates prepared from animals fed diets containing Dox (Fig. 3a).

To determine the levels of tau, a portion of these same brain homogenates was analyzed by immunoblot for tau (Fig. 3b), comparing mice on normal diets to mice that had been on the Dox diet for 7 days. From studies of the rTg4510 model of inducible tau expression, we expected that the levels of tau in mice fed Dox might not decline quickly due to the long in vivo half-life of tau (~11 days) [25]. As expected, immunoblots of these tissue homogenates showed that tau protein persisted longer than the luciferase protein, derived from the same mRNA (Fig. 3b). The blots revealed a substantial number of what appear to be breakdown products of the tau that may be a consequence of the presence of the c-terminal ubiquitin moiety that is fused to the tau sequence of the polyprotein (see Fig. 1). These data show that luciferase activity provides a much better report of transgene expression levels than the long lived tau protein. These data also reinforce the idea that significantly reducing steady-state tau levels will require sustained suppression of tau expression [23, 25].

## Discussion

Although is it quite feasible to determine the efficacy of gene knockdown in mouse models by directly assessing the levels of gene products, either mRNA or protein, the analysis of gene expression in the central nervous system (CNS) cannot be done in a longitudinal manner. For studies in which the goal is to optimize treatment regimens with gene silencing therapeutics, an ability to longitudinally assess target engagement in living animals would save both time and expense. Here, we describe the generation of transgenic mice expressing a tau-luciferase reporter built expressly for such a purpose. We included in the design of the model, the use of tetracycline-analog-responsive transgene vectors as a means to produce a positive control and assess the dynamic range of the luciferase reporter. Here we demonstrate the following: (1) the generation of a line of mice that express a tau-ubiquitin-luciferase fusion construct from a single mRNA; (2) the processing of this fusion protein to produce a tau protein that migrates to a size closely approximating the expectation for a tau-ubiquitin fusion protein; (3) high levels of bioluminescence in the brains of these mice when the tet-transactivator is co-expressed; (4) rapid decline in bioluminescence when transgene expression is silenced. Thus, this new model has several attributes that one would like to have in a model that is used to test gene silencing therapies targeting human tau.

We envision using this model to assess the efficacy of gene silencing therapies that target the gene at the level of the mRNA, such as ASOs, siRNA, and viruses encoding shRNAs. The model may also be useful in assessing therapies that target exonic sequences in the DNA directly, such as viruses encoding CRISPR/Cas9 targeting elements. Because the promoter elements that drive expression of the transgene are not derived from tau, the model cannot be used to screen drugs that specifically target transcriptional regulators of tau. Moreover, the model would not be particularly useful in screening drugs that target tau protein degradation. However, with a redesign of the cDNA, eliminating the ubiquitin element so that tau and luciferase remain attached as a fusion protein, it would be possible to create mice that could report tau protein levels. The model in its current form is also not appropriate for screening ASOs that would be designed to modulate splicing of tau pre-mRNA. However, with minimal re-designed an intron could be added to the construct to enable assessments of ASO efficacy in modulating splicing. This new model provides proof-of-concept demonstration of a means to produce a luciferase reporter mouse in which luciferase is expressed from an mRNA encoding tau while at the same time having the half-life of luciferase separated from the long-lived tau protein [25]. From prior studies of mice expressing tau from tetracycline-regulated vectors similar to what we have used here, we knew that the levels of transgene expressed tau mRNA fall rapidly when mice are treated with Dox [23]. The rapid decline in luciferase levels in the Dox-treated mice demonstrates that luciferase activity in this model is a good indicator of mRNA levels. Indeed, the rapid decline in bioluminescence activity in the bigenic tTA/TUL mice after suppressing transgene expression with Dox is typical for short-lived CNS proteins. For example, the levels of transgene expressed amyloid precursor protein in a tet-regulated model decline rapidly after mice are given food containing Dox [21]. Our data show that engineering an efficiently-cleaved proteolytic site between the target gene and luciferase provides an effective means to separate a luciferase reporter from a co-expressed gene of interest in vivo.

When we originally conceived of the transgene construct, we had some aspirations that the level of tau that would be produced by the TUL construct might rise to the level required to produced tau-mediated neurodegeneration, ultimately enabling a better prediction regarding the level of gene suppression that might be required to attenuate pathology. In hindsight, we now believe that absence of neurodegeneration in this model may be advantageous because neuronal loss would almost certainly cause the levels of luciferase to fall as cells expressing the transgene die. Studies in tetracycline-regulated tau mice have already demonstrated that silencing tau expression can be beneficial and that early intervention may be critical [23]. Our TUL mice provide a new approach to screen and optimize tau-silencing therapeutics that enables longitudinal assessments of target-engagement.

## Conclusions

In summary, we describe the production of a new model of human tau expression that we envision to be used in longitudinal assessment of tau-silencing therapeutics. In light of the current paucity of drug targets that could slow the progressive misfolding of tau as occurs in AD and other tau pathologies, targeting the production of tau has emerged as an attractive approach. The long half-life of the tau protein may be a serious barrier to this approach, requiring early intervention, but such a therapeutic paradigm may provide a ray of hope to patients at high risk of developing disease because they harbor disease-causing mutations in tau. Ultimately, as gene silencing therapeutics for CNS diseases move into the clinic with greater frequency, the risk-reward calculations for this invasive therapy may improve to the point of wider deployment in patients at high risk for all diseases in which tau pathology features prominently, such as AD.

## Abbreviations

NTg, non-transgenic mouse; tTA, tetracycline-transactivator; TUL, fusion protein tau-ubiquitin-luciferase
